# dendPoint: a web resource for dendrimer pharmacokinetics investigation and prediction

**DOI:** 10.1038/s41598-019-51789-3

**Published:** 2019-10-29

**Authors:** Lisa M. Kaminskas, Douglas E. V. Pires, David B. Ascher

**Affiliations:** 1 School of Biomedical Sciences, University of Queensland, St Lucia, Queensland Australia; 2 School of Computing and Information Systems, University of Melbourne, Melbourne, Victoria Australia; 3 Computational Biology and Clinical Informatics, Baker Heart and Diabetes Institute, Melbourne, Victoria Australia; 4 Structural Biology and Bioinformatics, Department of Biochemistry, Bio21 Institute, University of Melbourne, Melbourne, Victoria Australia; 5 Department of Biochemistry, University of Cambridge, Cambridge, Cambridgeshire United Kingdom

**Keywords:** Computational models, Databases, Machine learning

## Abstract

Nanomedicine development currently suffers from a lack of efficient tools to predict pharmacokinetic behavior without relying upon testing in large numbers of animals, impacting success rates and development costs. This work presents dendPoint, the first *in silico* model to predict the intravenous pharmacokinetics of dendrimers, a commonly explored drug vector, based on physicochemical properties. We have manually curated the largest relational database of dendrimer pharmacokinetic parameters and their structural/physicochemical properties. This was used to develop a machine learning-based model capable of accurately predicting pharmacokinetic parameters, including half-life, clearance, volume of distribution and dose recovered in the liver and urine. dendPoint successfully predicts dendrimer pharmacokinetic properties, achieving correlations of up to r = 0.83 and Q^2^ up to 0.68. dendPoint is freely available as a user-friendly web-service and database at http://biosig.unimelb.edu.au/dendpoint. This platform is ultimately expected to be used to guide dendrimer construct design and refinement prior to embarking on more time consuming and expensive *in vivo* testing.

## Introduction

A lack of appropriate pharmacokinetic behavior has historically been one of the leading causes of drug failure in clinical trials^1^. Advances in controlled release technologies and nanomedicine, however, are increasingly providing new opportunities to circumvent this shortcoming in rational drug development initiatives and are providing renewed hope for old drug candidates. Importantly, a wide range of nanosized materials with a variety of chemico-biological traits that can be used to alter and drive the pharmaceutical behavior of loaded drugs (including colloids, nanoparticles and polymers) have been developed and explored for their potential as relatively biologically inert drug carriers. One of the primary indications for which nanomaterials have been explored and have proven successful is in promoting the targeted delivery of chemotherapeutic drugs towards solid tumors via the enhanced permeation and retention (EPR) effect^2^. While optimal EPR necessitates the use of nanocarriers that display prolonged blood circulation^3^, a trade off needs to exist between blood exposure (to maximize EPR) and elimination (to minimize accumulation of the nanomaterial in the body and off-target toxicity). As a classic example, the PEGylated liposomal formulation of doxorubicin (Doxil®/Caelyx®) displays good EPR into solid tumors, but its prolonged plasma exposure leads to accumulation in the extremities, causing painful swelling of the hands and feet^4^. This highlights the importance of optimizing the pharmacokinetic behavior of nanomedicines early in development.

Often, however, the preclinical development of nanomedicines involves testing the biopharmaceutical behavior and safety of a wide range of nanocarriers, alone and in combination with loaded drug, in hundreds of animals prior to advancing the optimized construct(s) into clinical trials. This leads to increased research and development time and cost which, ultimately, translates into higher product costs for consumers. Furthermore, researchers are seeing an increasing impetus to limit the use of animals in biomedical research^5^. This has been addressed somewhat for small molecule drug candidates by the development of predictive models for toxico-pharmacokinetic behavior based on the physicochemical attributes of the drug (approaches such as pkCSM^6^). This enables preliminary *in silico* assessment of pharmacokinetic properties, guiding refinement of the molecule prior to *in vivo* testing. To date, however, no such predictive models exist for macromolecules and nanomaterials. This is in part due to the wide diversity in available nanostructures that can be employed as drug delivery systems, with each displaying distinct *in vivo* behavior. Even within defined classes of nanomaterials, changes to the nanomaterial composition, drug loading, length and number of surface polyethylene glycol (PEG) groups, for instance, can have profound and, until recently, seemingly unpredictable effects on biopharmaceutical behavior by altering the solution behavior and cell/protein binding properties of the material^7^. This is especially problematic for polymer-based systems (linear and hyperbranched polymers) which are typically much smaller (≤20 nm or <500 kDa) than colloids and nanoparticles (typically > 100 nm) and are therefore, more sensitive to small changes in composition and physicochemical properties.

In an attempt to address the lack of effective predictive models for the *in vivo* behavior of nanomaterials, Riviere and colleagues^8^ published the first approach to predict the adsorption of biomolecules onto a nanoparticle surface in Nature in 2010. The approach involved comparing the surface adsorption of a set of small molecule probes and generating a ‘surface adsorption index’ to predict the binding of biomolecules (the ‘protein corona’) which is known to play a significant role in dictating the biodistribution behavior of nanoparticles^9^. Subsequent to this, a number of investigators have used physiologically based pharmacokinetic models (PBPK) to simulate the mass-time biodistribution profiles for a range of metal nanoparticles^10–15^ as well as some polymeric nanoparticles^16–18^. In most cases, these models were developed based on limited experimental data sets to predict the biodistribution and elimination kinetics of nanoparticles with a fairly narrow set of physicochemical variants (such as size and charge). The intention behind these models was to aid researchers in their selection of optimal particle properties for further development or in risk assessment analysis. The PBPK approach however, is not appropriate for predicting the pharmacokinetic behavior of more complex nanostructures such as liposomes and polymers that may be comprised of a variety of different scaffold components (such as different lipids or monomers). These models are also not easily adaptable and available for use by researchers with limited or no knowledge of biometric analysis.

**Figure 1:**
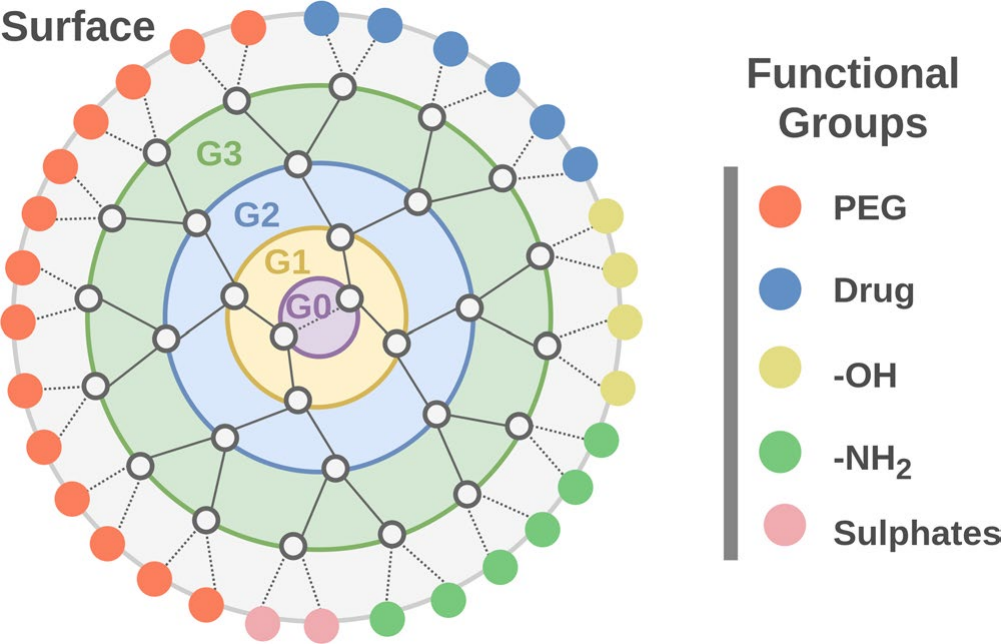
Basic structure of a dendrimer showing sequential layering of monomeric units around a central core (G0). A dendrimer may be composed of any monomeric unit provided it has at least 2 functional groups available to build additional generations. Surface functional groups depicted as circles.

Dendrimers are well defined hyperbranched polymeric systems that can range in size from 1–20 nm in diameter^19^ (Fig. 1), which can provide several pharmacokinetic advantages over much larger colloids and nanoparticles^20–22^. Drugs can be loaded either peripherally via internally triggered chemical linkers, or can be non-covalently loaded into the hydrophobic scaffold. Although the clinical advancement of nanomedicines has been a slow process, Starpharma’s topical microbicidal gel (Vivagel®) has recently gained regulatory approval in Australia and Europe for the treatment of bacterial vaginosis and a dendrimer-based formulation of docetaxel (DEP™-docetaxel) recently successfully completed phase I clinical trials for the treatment of advanced solid tumors. The establishment of an *in silico* model capable of accurately predicting dendrimer pharmacokinetics is therefore timely and of increasing relevance.

Here, we describe dendPoint, the first *in silico* and widely available model to predict the intravenous pharmacokinetics of complex polymeric nanomaterials based on scaffold structure and physicochemical properties. We have manually curated a detailed relational database describing dendrimer biopharmaceutical behavior with various structural and chemical characteristics. This was used to develop a model to predict key pharmacokinetic parameters for dendrimers. dendPoint is available via a user-friendly freely available web-based system, accessible at http://biosig.unimelb.edu.au/dendpoint. This computational platform encompasses a relational database of pharmacokinetic properties of different dendrimer scaffolds together with a web-service capable of predicting and comparing dendrimer properties, including Half-life, Volume of Distribution, Clearance and Dose in Liver and Urine, allows users to rapidly and easily browse literature-derived properties as well as predict, compare and visualize dendrimer pharmacokinetic properties.

## Results

### Database curation

**Figure 2:**
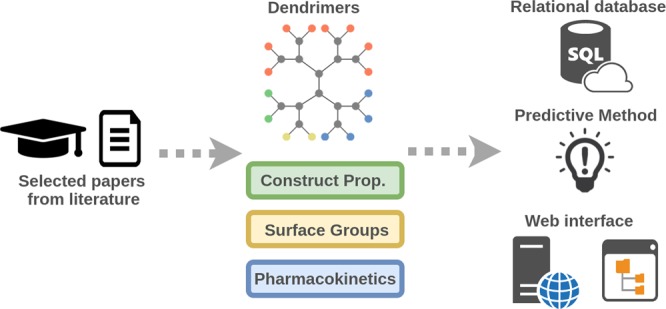
dendPoint workflow. Dendrimer pharmacokinetics were collected from over 600 papers via literature search. Construct properties, information on surface functional groups as well as pharmacokinetic behaviour of 69 different dendrimers were collected and included in a relational database. This was used as evidence to train and test predicted methods via supervised learning. A user-friendly web interface was created for both database and predictive method.

In total, the pharmacokinetic parameters of 69 distinct dendrimers, from over 600 papers, were manually curated into the dendPoint database (Fig. 2, Table S1). Many structural and physicochemical properties can dictate the pharmacokinetic behavior of dendrimers, including scaffold composition, dendrimer size, degree of surface PEGylation, PEG chain length, surface functionality (including charge and the presence of hydrophobic drugs) and structural flexibility^3,23,24^. The impact of each of these parameters on intravenous pharmacokinetics has been summarized previously^3^ and they were therefore included in the database as summarized in Table S1 (Supporting Information).

The susceptibility of the dendrimer scaffold to *in vivo* biodegradation may also impact upon the rate of dendrimer elimination from the body, but surface functionalization with non-biologic groups (such as non-natural amino acids and PEG) slows scaffold breakdown^3^. To this end, with the exception of two unmodified amine-terminated polylysine dendrimers, none of the dendrimers in Table S1 were reported in their respective publications to have shown significant *in vivo* biodegradation that was expected to have driven the reported intravenous pharmacokinetics. Biodegradability of the scaffold was, therefore, not included as a parameter that defined the ultimate pharmacokinetics, although the composition of the scaffold was.

Pharmacokinetic data was therefore obtained for dendrimers based on non-biodegradable triazine and polyamidoamine (PAMAM) scaffolds, as well as biodegradable polyester and polylysine scaffolds. While the effective ‘size’ of polymeric nanomaterials may be reported in terms of hydrodynamic radius or molecular weight, few of the papers reported in Table S1 provided this information on radius and therefore, this parameter could not be included in the database. Regardless, it has previously been suggested that the terminal Half-life of dendrimers correlates more significantly with molecular weight than with radius^3^. In addition, three quarters of the dendrimers included in dendPoint presented some degree of surface PEGylation. This is likely a result of the fact that since the molecular weight of the dendrimer scaffold is limited by poor conjugation efficiency and greater polydispersity for generations higher than approximately 5–6, surface PEGylation is commonly required to increase size and prolong plasma exposure^3^. PEGylation is also often employed either alone or in combination with acetylation (Ac) to block surface reactive sites and prevent binding to cells and tissues.

Pharmacokinetic parameters that were included in the database include terminal Half-life and clearance, since these ultimately describe the plasma exposure of the dendrimers after intravenous delivery. From these parameters, volume of distribution can be calculated from the equation *Cl* = Vd*(0.693/*t*_1/2_), where Cl denotes Clearance, *Vd* denotes terminal Volume of Distribution and *t*_1/2_ denotes Half-life. The percentage of the dose excreted via the urine and percentage dose recovered in liver were also included since these represent the major pathways by which dendrimers are cleared from plasma (*i.e*., via elimination in urine and biodistribution towards the liver). For liver uptake and urinary excretion, a threshold of <20% was used in classification tasks to define dendrimers with limited liver uptake or urinary excretion respectively. With the exception of several polyester dendrimers, significant quantities of dendrimer have not been detected in the feces, suggesting this is not a major route of dendrimer elimination from the body. Liver biodistribution typically results from recognition of the polymeric nanomaterial by macrophages of reticuloendothelial organs (which also include the spleen, lymph nodes and lungs) and is normally the organ that contains the highest proportion of an injected dendrimer dose after one week^3^. Distribution towards the liver may result from initial plasma protein binding (opsonisation) of the dendrimer, electrostatic recognition of anionic charges on the dendrimer surface by macrophages or non-specific accumulation of long circulating constructs over time^23–25^.

### Analysis of dendrimer properties

The distribution of all four experimental pharmacokinetic properties for all molecules in the database are shown in Fig. S1. This highlights the relatively broad distribution of Half-life, Clearance and percentage dose recovered in the urine across the range of dendrimer constructs that have been characterized. There was little accumulation of dendrimer dose (median of 7%) in the liver for the majority of constructs in the database (with 80% of dendrimer having a %Dose in Liver below 20%).

**Figure 3:**
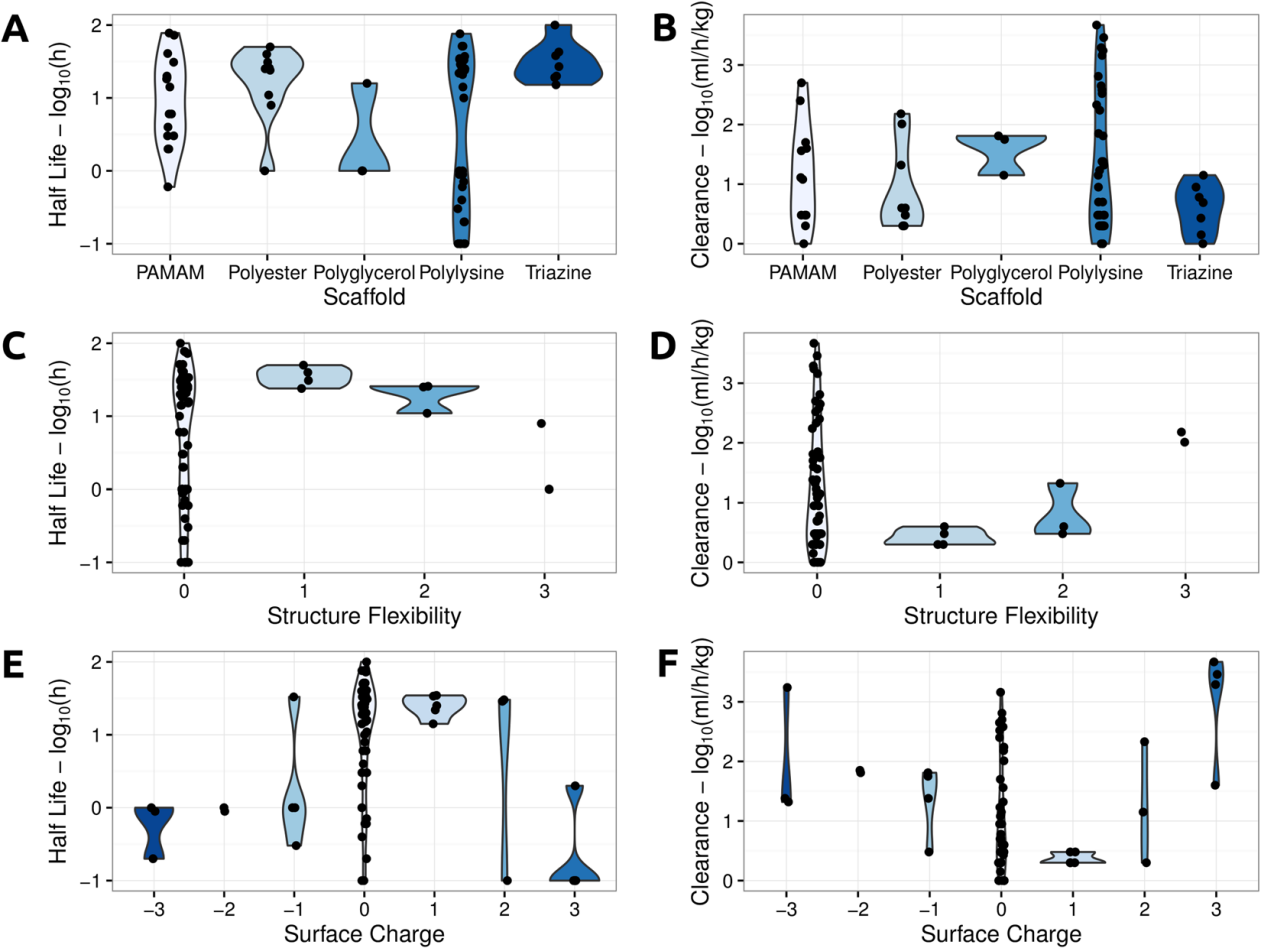
Distribution of Half-life and Clearance properties based on Scaffold, Structure Flexibility and Surface Charge across the database. The left-hand side graphs depict (from top to bottom), as violin plots, the distribution of Half-life per Scaffold, Structure Flexibility and Surface Charge, while the right-hand side graphs show how Clearance varies based on these properties.

Looking closer at the distribution of Half-life and Clearance divided by Scaffold and Flexibility revealed some general trends (Fig. 3A–D). Notably, as structural flexibility increased, Half-life decreased and Clearance increased. When assessing the effects of Surface Charge on the pharmacokinetic properties (Fig. 3E,F) a similar behavior was also observed. While Clearance increased as Surface charge move further from neutral (increasing charges, either negative or positive), Half-life decreased. The polylysine-based scaffold presented the largest variability in pharmacokinetic properties, in part due to the large number of diverse constructs that have been systematically analyzed to date. Triazine-based dendrimers were associated with lower Clearance and longer Half-lives.

**Figure 4:**
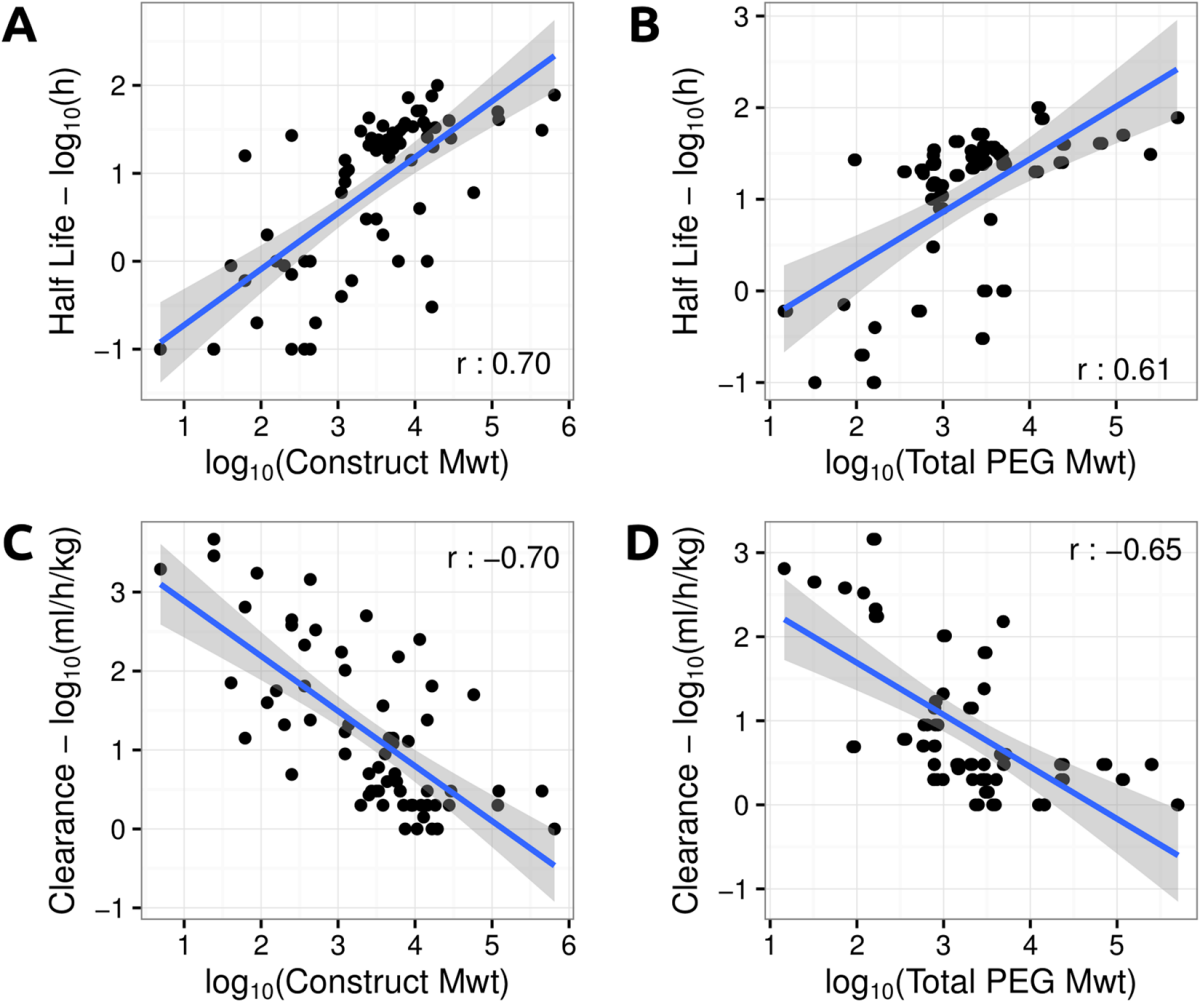
Distribution of Half-life and Clearance properties based on Construct and Total PEG molecular weight. The left-hand side graphs depict, as scatter plots, the distribution of Half-life (top) and Clearance (bottom) based on Construct molecular weight, while the right-hand side graphs show how these properties vary based on Total PEG molecular weight. Pearson’s linear correlations between these properties are also depicted.

PEGylation level and construct molecular weight correlated well with all pharmacokinetic properties. Half-life significantly and positively correlated with both construct molecular weight (r = 0.70) and total PEG molecular weight (r = 0.61) (Fig. 4A,B). An even stronger correlation, although negative, between these properties and Clearance was also observed (r = −0.70 for construct molecular weight and r = −0.65 for total PEG molecular weight) (Fig. 4C,D).

### Prediction of dendrimer pharmacokinetic properties

Our curated database was then used to train and test predictive pharmacokinetic models. The approach we used was based on our well validated CSM methodology^26^. This is based on the concept of structural signatures, which are an alternative way of extracting relevant patterns from molecular entities, originally modeled as graphs, which in turn are provided as evidence to supervised learning methods. These structural signatures are a powerful and scalable way to represent geometry and physicochemical properties, and have been applied to accurately predict small molecule pharmacokinetics^6,27^, to characterize small molecule-protein interactions^28^ and the effects of mutations on protein structure^29–37^. As shown in Fig. 1, we represent each dendrimer as a graph where the nodes are the branch points and the edges are the connections. Distance patterns between nodes are then summarised as cumulative distribution functions, which are then used as evidence to train machine learning methods. Complementary information also integrated into the signatures included the physicochemical properties in the curated database. This information, together with the experimentally measured pharmacokinetic properties, was then used to train and test predictive models.

**Figure 5:**
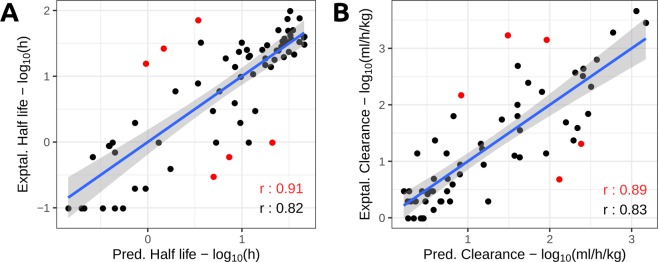
Predicting dendrimer pharmacokinetcs with dendPoint. The graphs depict the correlation between experimental and predicted properties on jackknife validation. A correlation of r = 0.82 was obtained when assessing Half-life, increasing to r = 0.91 when 10% outliers (shown in red) were removed. While predicting Clearance, dendPoint achieves a correlation of up to r = 0.89 on 90% of the data.

The dendPoint platform for predicting dendrimer pharmacokinetic properties was able to accurately predict Half-life with a Pearson’s correlation coefficient of r = 0.82 and Q^2^ = 0.66 on jackknife validation (Table 1, Fig. 5A). This correlation increases to r = 0.91 when assessing the performance of the method after removing 10% of outliers. A similar performance, however with smaller dispersion, was observed for the Clearance predictor. A correlation of r = 0.83 and Q^2^ = 0.68 was obtained on cross validation, also increasing to r = 0.89 after 10% outlier removal (Table 1, Fig. 5B). To further evaluate the predictive models, we assessed their performance on a bootstrap validation using a 90%/10% split over 100 repetitions. The performance of all models was consistent with that achieved over jackknife validation as shown in Table 1.

**Table 1:** dendPoint evaluation on jackknife cross validation, and 100 times bootstrap validation on a 90%/10% split with 100 repetitions.

Data set	Jackknife Cross Validation		Bootstrap	
	Pearson’s	RMSE	Pearson’s	RMSE
Half-life	0.82	0.52	0.76	0.54
Clearance	0.83	0.57	0.82	0.54
%Dose Liver	0.59	15.7	0.57	13.6
%Dose Urine	0.73	15.5	0.70	14.2

For both Half-life and Clearance predictors, the outlier sets were composed, on average, of smaller constructs (Construct molecular weight of Half-life outliers = 33.8 kDa, and Clearance outliers = 17.2 kDa, compared to the average construct molecular weight across the dataset of 47.9 kDa), with less surface PEGs (Half-life outliers = 8.5, Clearance outliers = 6.4 compared to 16.2 average surface PEGs across the dataset), accounting for a smaller total PEG molecular weight (Half-life outliers = 15.9 kDa, Clearance outliers = 11.5 kDa compared to the overall dataset that had an average total PEG molecular weight of 30.3 kDa).

Building a predictor for %Dose in liver presented a great challenge given the very skewed distribution of experimental values, as seen in Fig. S1C. Despite the skewed distribution of experimental data, dendPoint was able to achieve a correlation of r = 0.59, which increased significantly once the top 10% of outliers were removed, reaching a correlation of r = 0.73 on 90% of the data (Table 1, Fig. S2A). Predictive performance for %Dose Liver did deteriorate slightly for larger predicted values due to the skewed nature of the data distribution.

The predictor for %Dose in urine, achieved a higher correlation in comparison with % Dose in Liver, with a correlation of r = 0.73, increasing to r = 0.87 after 10% outlier removal (Table 1, Fig. S2B). This is largely due to the data of %Dose recovered in the urine within the dendPoint database that had a more even distribution of experimental values. Bootstrap validation of both %Dose in liver and %Dose in urine showed consistent performance to jackknife (Table 1), further improving confidence in both models predictive capabilities.

Alternatively, we were also able to build predictors to assess whether a dendrimer construct would be cleared by the liver and/or excreted in urine. Constructs were defined as having limited liver uptake or urinary excretion using a 20% cutoff for dose in liver and urine, respectively (see details in Materials and Methods). Figure S3 of Supplementary Materials shows the ROC curves obtained for both predictors, which achieved AUCs of 0.87 and 0.86 for urinary excretion and liver uptake, respectively. The predictors were successful in predicting the differences between constructs that had limited liver uptake/limited urinary excretion compared to those that were cleared by the liver or excreted in urine, achieving an accuracy of up to 80%.

The attributes that contributed most to the performance of each predictor were evaluated by Principal Component Analysis (PCA). Both construct molecular weight and total PEG molecular weight were consistently well ranked for all four predictors. This was expected given these attributes correlated well with the pharmacokinetic parameters by themselves. PCA showed that these attributes contributed largely to the variability of the Half-life data set, together with Generation and Structure Flexibility (Fig. S4A). These were consistent with the other data sets. Drug conjugation, both in terms of the type and number of surface drugs, played an important role in prediction performance, despite a clear correlation not being noticed during analysis of individual features. A histogram of the percentage of explained variance per feature (Fig. S4B) shows that the majority of the features are necessary to explain variability (a linear drop on explained variance, instead of a usual logarithmic drop), suggesting that the selected group of variables are diverse and complementary.

## Discussion

In summary, here we describe dendPoint, the first relational database and predictive method that associates physicochemical properties of a complex hyperbranched polymeric structure (notably dendrimers) with experimentally measured intravenous pharmacokinetic data. This provides the first opportunity to begin to systematically analyze the relationship between dendrimer structures and their biological behaviors, in the attempt to guide construct design and development. It has been carefully curated from the literature and will be updated regularly. Although, in practice, dendrimers may ultimately be delivered via non-intravenous routes (such as subcutaneously or via inhalation) which will require the need for additional base physicochemical properties for optimal pharmacokinetic behavior, the intravenous route is currently standard practice for the systemic delivery of nanomedicines.

This database reveals some general rules of dendrimer design, where construct molecular weight, flexibility and PEGylation can all be used to tunably adjust the plasma exposure of a dendrimer. To begin with, whilst half-life is used as the most common parameter to describe the plasma exposure of a nanomaterial, clearance is a more appropriate parameter since it takes into account urea under the whole plasma concentration-time profile, rather than simply the elimination kinetics alone. With this in mind, plasma exposure can be increased (or rather, clearance decreased) via restricting urinary excretion, extravasation and uptake into cells and tissues by employing the following basic rules: (1) increased degree of surface conjugation or molecular weight of hydrophilic, biocompatible and poorly-biodegradable polymers such as PEG (polymerisation), (2) increase construct molecular weight (size), (3) reduce surface charge (charge), (4) reduce structural flexibility (flexibility). Based on the available data and our model, drug conjugation to the surface has a negligible effect on intravenous pharmacokinetics.

In small molecule drug design, the development and application of generalized rules and these tools have been widely used to improve compound quality and success rates. In addition to providing the first curated database of dendrimers to facilitate the analysis of nanoparticles, we demonstrate that it can be used as the basis to train novel predictive pharmacokinetic models.

We have implemented a user-friendly web server that will enable researchers to search, predict and visualize the pharmacokinetic properties for their molecules of interest (http://biosig.unimelb.edu.au/dendpoint). In addition, we have implemented a comparison feature that enables users to rapidly compare the pharmacokinetic profiles of two molecules, allowing systematic evaluation of pharmacokinetic profiles as various physicochemical properties of the dendrimer are modified. Considering the sensitive nature of many projects, the web server does not retain any information submitted to it. This will hopefully facilitate the development and optimization of dendrimers for specific biological roles, and provide a foundation for the evaluation of nanoparticles more broadly.

## Methods

### Collation of published pharmacokinetic data

Published work describing the intravenous pharmacokinetics of dendrimers were identified by undertaking PubMed searches of the terms ‘dendrimer and pharmacokinetics’ or ‘dendrimer and biodistribution’. Only papers that described - or provided enough information to extrapolate – composition of the dendrimer scaffold and surface, molecular weight, terminal plasma half-life (*t*_1/2_) plus % dose excreted in urine and/or % dose recovered in liver at termination were included in the database. Papers describing the pharmacokinetics of loaded drug rather than the dendrimer scaffold were excluded, since following the loaded drug is a poor predictor of the biopharmaceutical behavior of the dendrimer itself. Supramolecular structures (such as micelles) that were comprised of dendritic polymers and hyperbranched polymers that were based on dendritic cores were also excluded. Dendrimers conjugated with distinct targeting moieties (such as tumor targeting ligands) were also not included since this is expected to change the pharmacokinetics of the base construct.

Using this criteria, 20 papers collectively describing the pharmacokinetics of 69 distinct dendrimer structures were identified that provided sufficient information to compile the database. Plasma clearance (*Cl*) was included in the database where possible and was either taken directly from published papers or was extrapolated from available data by dividing dose by area under the plasma concentration versus time curve. Where pharmacokinetic parameters needed to be extrapolated from graphical data, the data recovery program GetData Graph Digitizer v2.26 (GetData Pty Ltd, NSW, Australia) was employed. Where area under the plasma concentration time curve needed to be calculated to determine *Cl*, this was calculated manually using the trapezoid rule and extrapolated to infinity by dividing the last plasma concentration detected by the elimination rate constant (*k*).

Each of the papers used described dendrimer pharmacokinetics in rodent models (notably mice and rats), and as such, the body weight of the animal models used can vary by up to 20 fold. While this has no bearing on *t*½ or proportion of injected dose recovered in liver or urine, *Cl* is a function of distribution volume (*Vd*) and therefore body weight. *Cl* was therefore normalized to ml/h per kg body weight by dividing *Cl* (in ml/h) by the average reported body weight of animals used in the study. Where mean body weight or body weight ranges were not reported, mean body weights for the animals described and at the reported ages were extrapolated from growth curves published online by several breeders (Harlan, Charles River Laboratories, Taconic and the Animal Resources Centre).

The complete set of data used to compile the pharmacokinetic database is tabulated in Table S1 (see Supporting Information).

### Method training and evaluation

Different supervised learning algorithms for regression available on the Weka ToolKit (version 3.8.2)^38^ were used to train models for the different pharmacokinetic properties collected and store on the database. The best performing models, based on the evaluation metrics below, were obtained using Random Forest^39^ (default settings, 100 trees) for both classification and regression tasks.

Regression models were initially evaluated using jackknife validation procedure and the best performing models were selected based on the Pearson’s Correlation Coefficient and Root Mean Square Error (RMSE; $RMSE=\sqrt{\mathop{\sum }\limits_{i=1}^{N}\,\frac{{(Predicte{d}_{i}-Actua{l}_{i})}^{2}}{N}}$). The Pearson’s Correlation Coefficient quantifies the linear dependency between two variables (e.g., experimental vs. predicted pharmacokinetic properties) as the covariance of the variables divided by the product of their standard deviations: *r*_*A,B*_ = *cov*(*A,B*)/*sd*(*A*)∗*sd*(*B*). Correlations vary from [−1, 1], where 1 and −1 denote a perfect positive and negative linear correlation, respectively, while a correlation value of 0 denote no linear correlation. Classification models were also evaluated using jackknife validation and best performing ones selected based on Area Under ROC curve (AUC) and accuracy. AUC varies from [0, 1] with a random binary classifier achieving an AUC = 0.5 and a perfect classifier achieving an AUC = 1. Regression and classification models were further evaluated using bootstrap validation on a 90%/10% split of the data, with 100 repetitions. Outliers are considered the points furthest away from the line of best fit, and were removed only for analysis purposes. Accuracy is denoted by the proportion of correctly classified instances. PCA was performed using R programming language to assess each feature’s contribution to explain variability.

### Database and web interface

dendPoint’s database and predictive models have been implemented as a user-friendly web-server freely available at http://biosig.unimelb.edu.au/dendpoint. The dendrimer pharmacokinetics information collected from literature search was consolidated as a MySQL relational database (version 5.5.35). Front-end development was created using the Bootstrap framework (version 3.3.7). The server back-end runs on a Linux server and was implemented in Python using the Flask frame-work (version 0.12.3). Dendrimer depiction was developed with the JavaScript library D3.js (version 4.2.6) and Plasma Concentration plot was created with the HighCharts charting tool (version 5.0.0).

Users have the option to browse the database via the web interface (Fig. S5, Supporting Information), search/filter specific information as well as show/hide construct properties, surface group properties and phamacokinetics details.

Job submission can be easily done by informing construct properties and surface group compositions via an intuitive submission form (Fig. S6). Generation and Construct molecular weight are required fields. After prediction a results page is exhibited (Fig. S7), showing the pharmacokinetics properties, dendrimer depiction and plasma concentration curve, giving the user the option to wither modify the job for resubmission or compare the predicted properties with another dendrimer construct (Fig. S8). See Supporting Information for supplementary figures.

## Supplementary information

Supplementary Info
