# Supplementary material for: dendPoint: a web resource for dendrimer pharmacokinetics investigation and prediction
Source: Sci Rep. 2019 Oct 29;9:15465. doi: 10.1038/s41598-019-51789-3 (PMC6820739; doi:10.1038/s41598-019-51789-3)
Supplement: Supplementary file 1 — Supplementary Info [file 41598_2019_51789_MOESM1_ESM.pdf]

Supplementary Materials

dendPoint: a web resource for dendrimer pharmacokinetics investigation and prediction

Lisa M. Kaminskas[a,†,*], *Douglas E.V. Pires*[b,c,d,†], *David B. Ascher*[c,d,e,*]

[a] School of Biomedical Sciences, University of Queensland, St Lucia, Queensland, Australia;

[b] School of Computing and Information Systems, University of Melbourne, Melbourne, Victoria, Australia;

[c] Computational Biology and Clinical Informatics, Baker Heart and Diabetes Institute, Melbourne, Victoria, Australia

[d] Structural Biology and Bioinformatics, Department of Biochemistry, Bio21 Institute, University of Melbourne, Melbourne, Victoria, Australia

[e] Department of Biochemistry, University of Cambridge, Cambridge, Cambridgeshire, United Kingdom;

†These authors contributed equally.

*To whom correspondence should be addressed. D.B.A. Tel: +61 90354794; Email: david.ascher@unimelb.edu.au; Correspondence may also be addressed to L.M.K: l.kaminskas@uq.edu.au

Supplementary Methods

Machine learning algorithms

Random Forest is an ensemble supervised learning method that is based on the construction of a number of small/simple base predictors using decision trees (forest), outputting the average prediction in case of regression tasks or the mode in case of classification.

Supplementary Figures

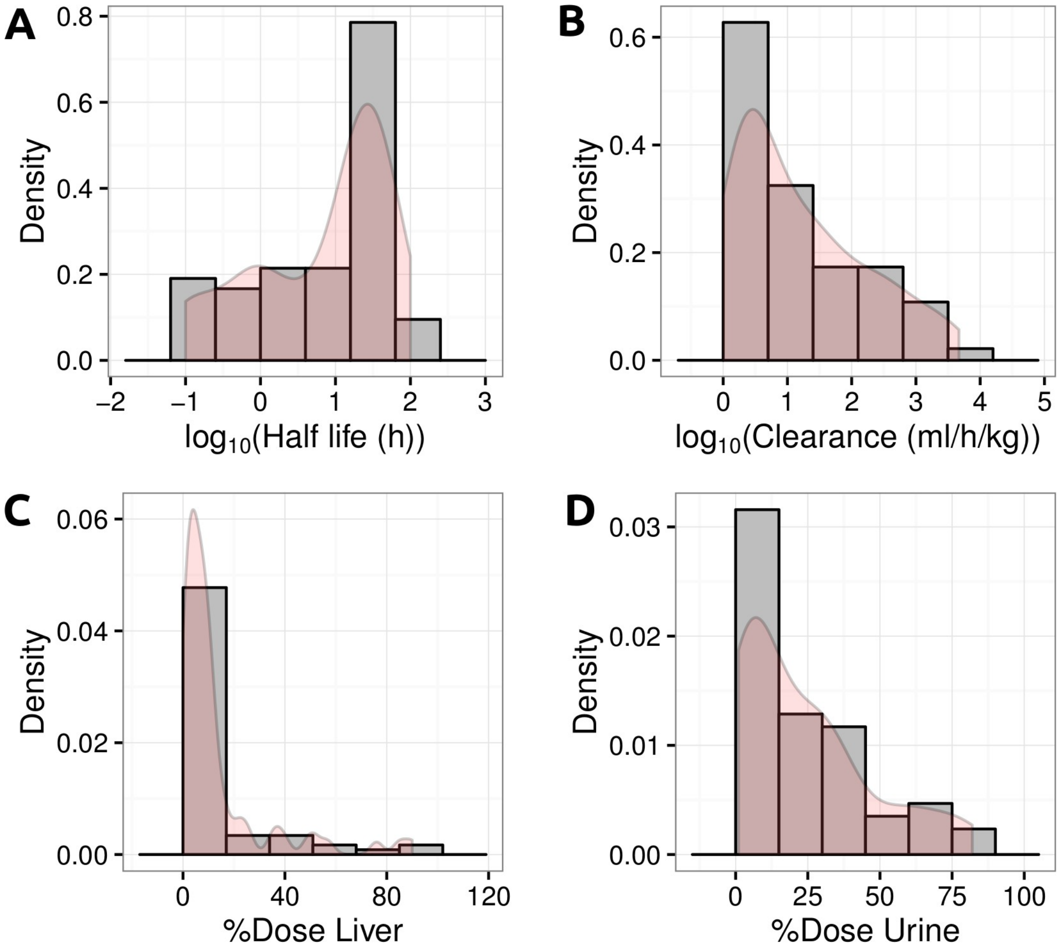

Figure S1: Distribution of experimental pharmacokinetics parameters for dendrimer constructs on the database.

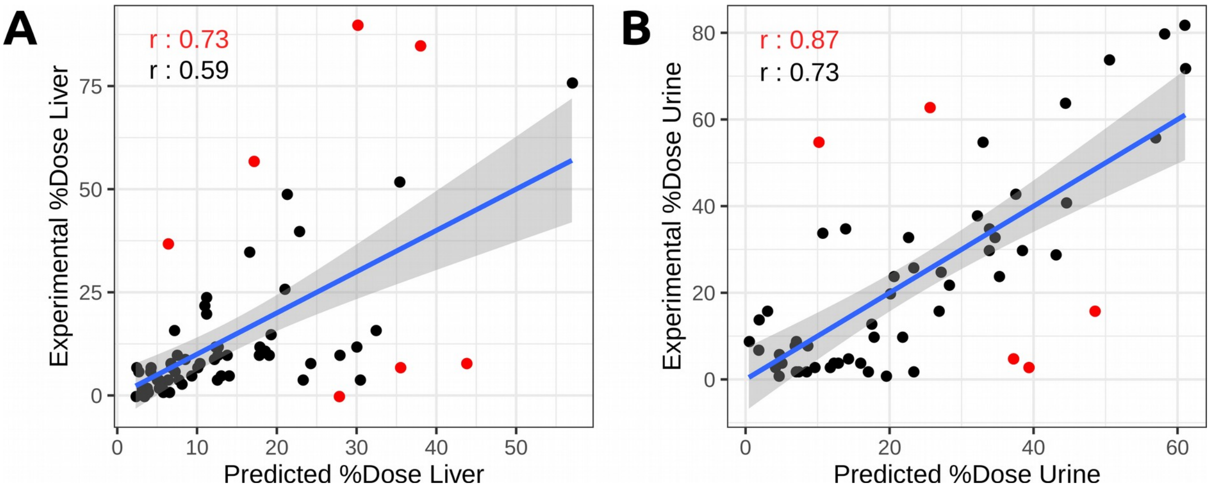

Figure S2: Predicting the percentage of dose that is recovered in the liver and urine. The graphs depict the regression plot between experimental and predicted %Doses for Liver (left-hand side graph) and Urine (right-hand side graph), which obtained of up to r=0.87 after 10% outliers were removed (shown in red).

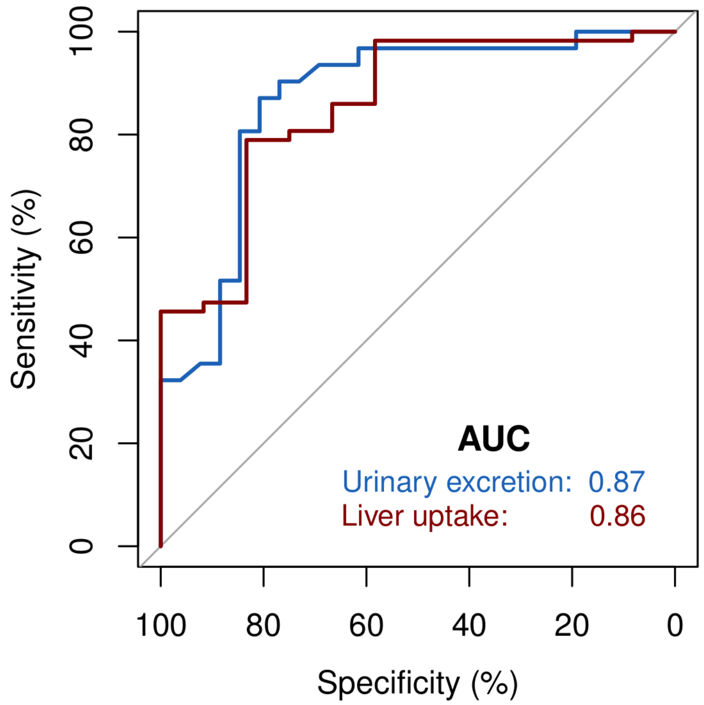

Figure S3: ROC curves dendrimer construct classification according to liver uptake and urinary excretion. Both predictions achieved an accuracy of 80% on these tasks, achieving AUCs of 0.87 and 0.86 for urinary excretion and liver uptake, respectively.

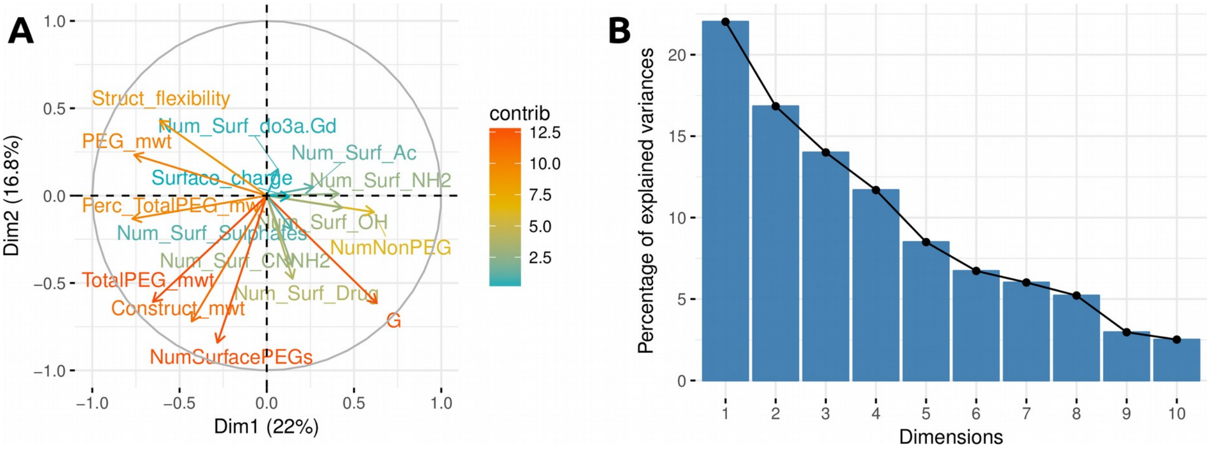

Figure S4: PCA analysis for he Half Life data set. The left-hand figure depicts the contribution of each feature to explain the variability of the data set. The right-hand figure shows a histogram of the percentage of explained variance per feature.

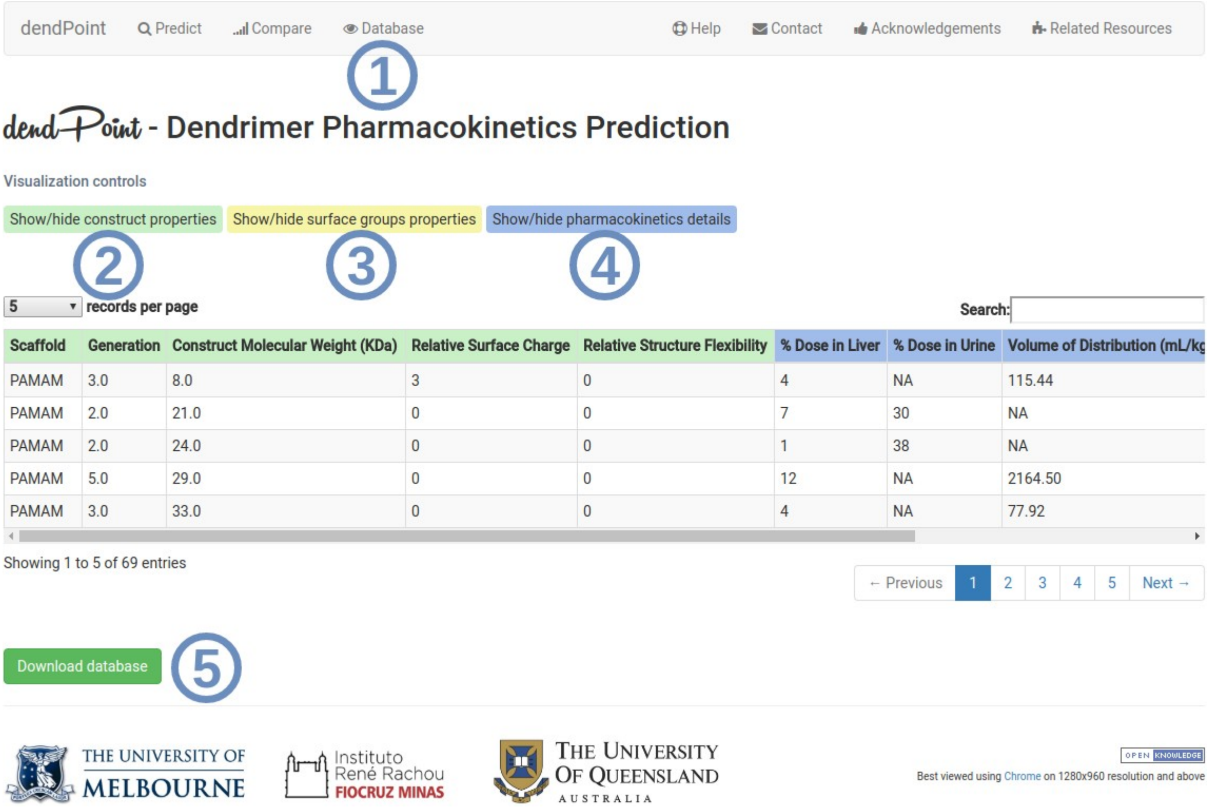

Figure S5: dendPoint database. The figure depicts the web-based interface for browsing the relational database linking dendrimer properties and pharmacokinetic behavior. By accessing the browsing option (1), users have the option to show/hide different properties (2,3,4) as well as download the full contents of the database (5).

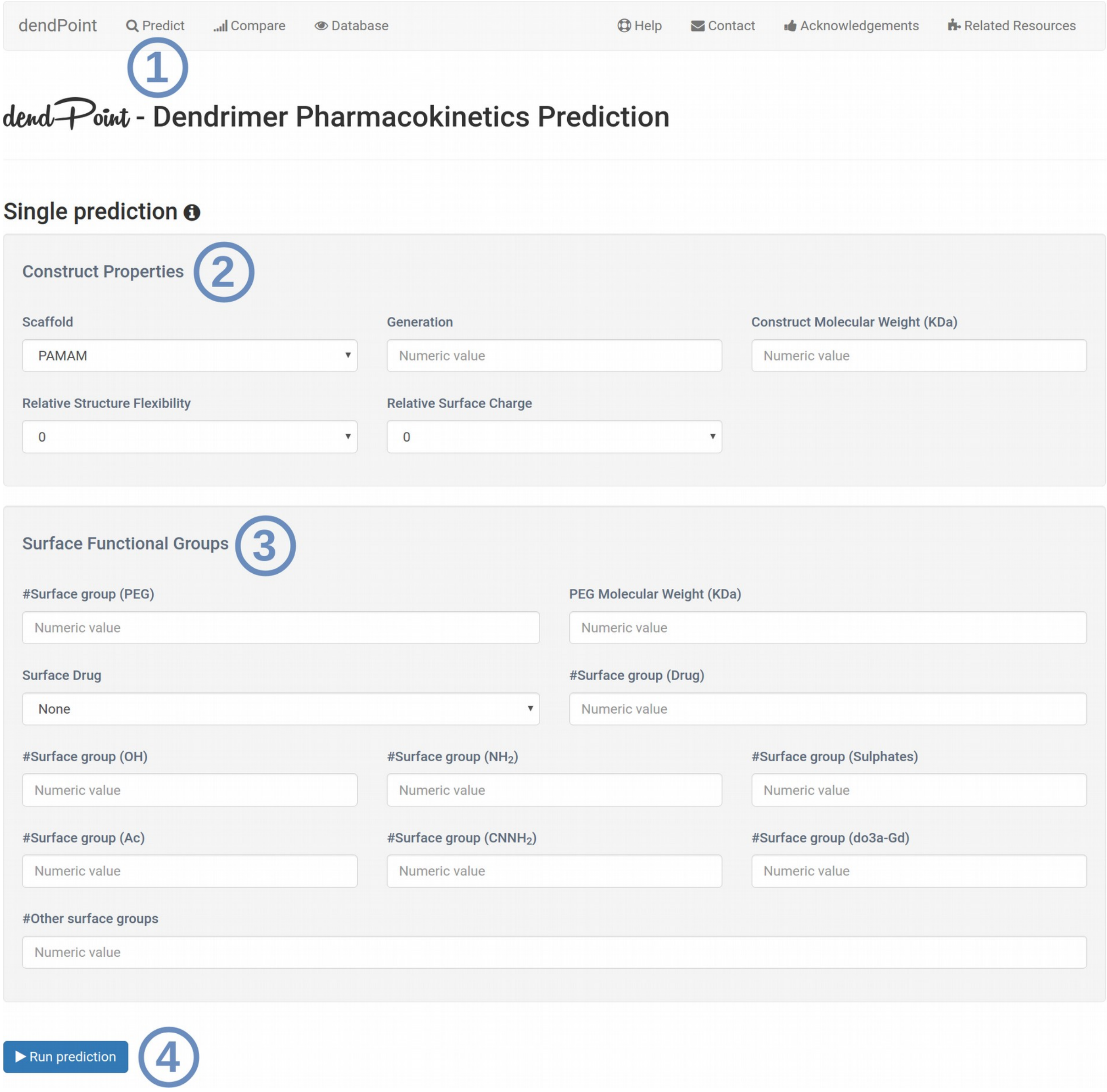

Figure S6: dendPoint submission page. The figure depicts the web-based interface for job submission. By selecting the prediction mode (1), users can specify different construct properties (2) and surface functional groups (3) prior to submission (4).

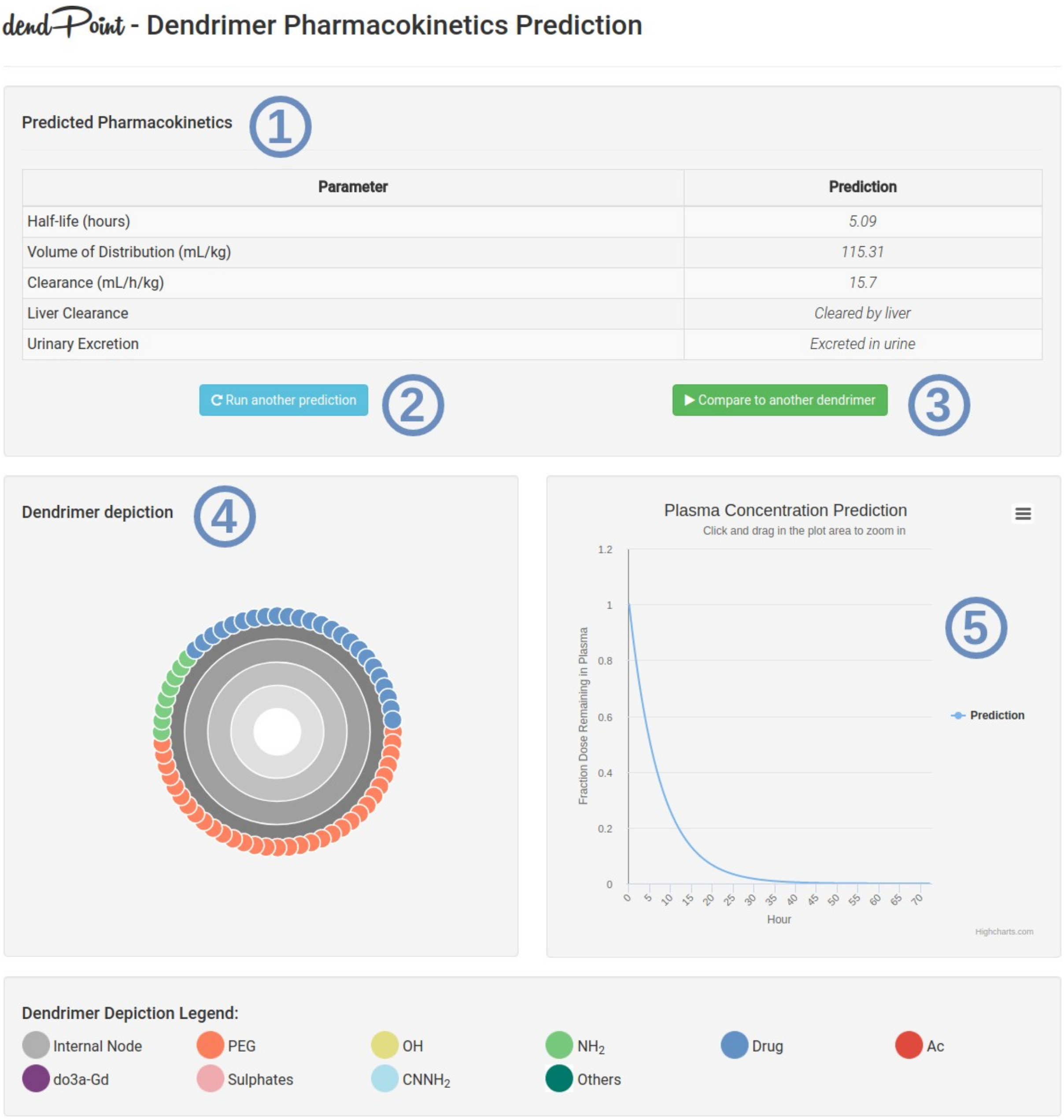

Figure S7: dendPoint result page for single dendrimer predictions. The figure depicts the prediction result page for a single dendrimer. The predicted pharmacokinetic properties for a user-defined dendrimer construct are exhibited in tabular format (1). The interface gives the user the option to either run another prediction (2) or compare the current construct with another one (3). Also a dendrimer depiction is available, showing the number of generations (as concentric grey circles) and surface groups as spheres. A plasma concentration prediction curve is also provided (5).

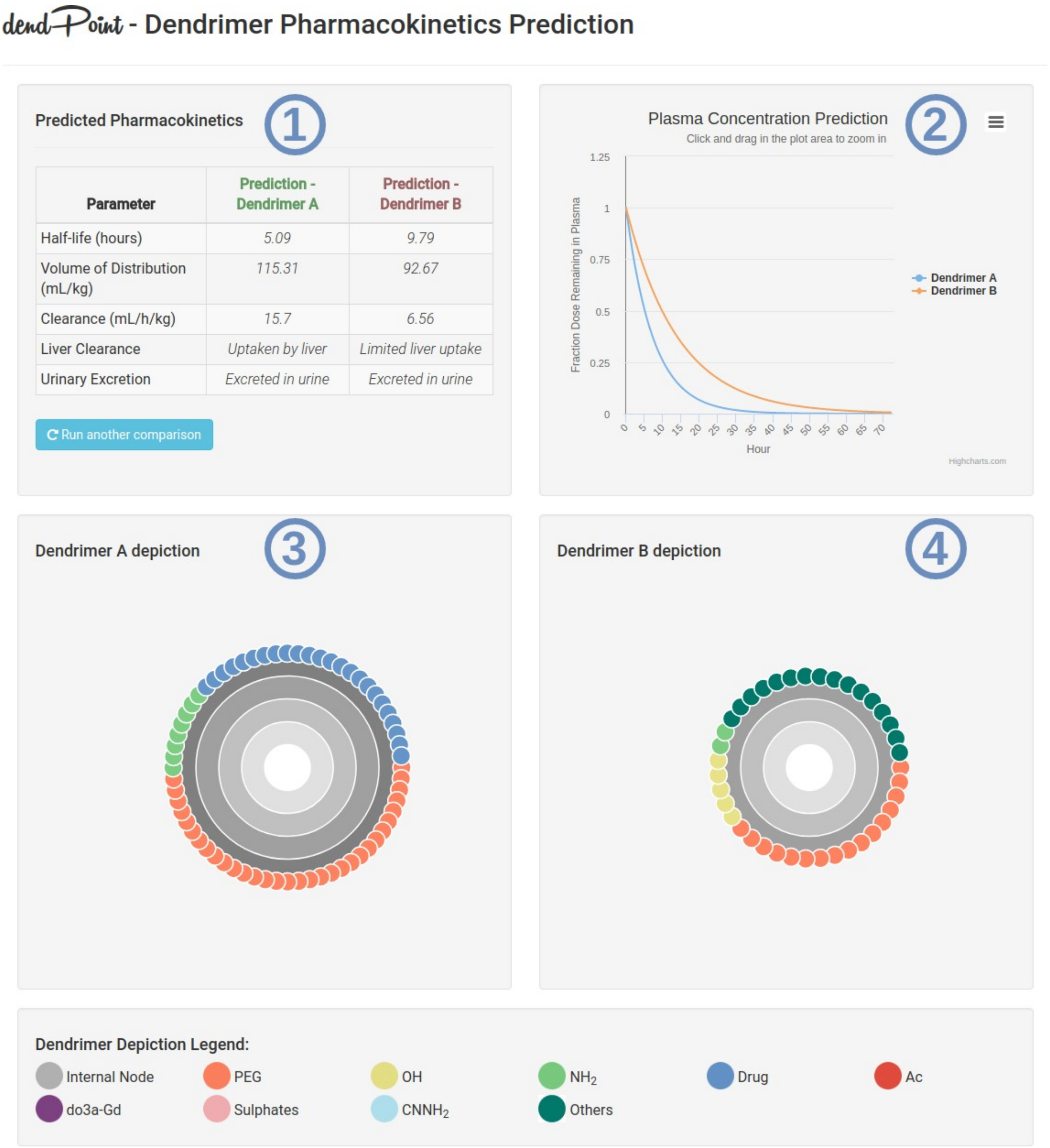

Figure S8: dendPoint result page for dendrimer comparison. The figure depicts the prediction result page for comparing pharmacokinetics of two dendrimers. The predicted pharmacokinetic properties are exhibited in tabular format for both dendrimers (1). The plasma concentration prediction curves for both constructs are also provided (2). The dendrimer depictions are plotted side-by-side, showing the number of generations (as concentric grey circles) and surface groups as spheres (3-4).

Table S1. Summary of structural characteristics and pharmacokinetic properties for dendrimers that were included in the database.

Scaffold	G[a]	# Surface PEG (kDa)	# non-PEG sites	# non-PEG surface functionality[b]	Surface drugs[c]	Surface charge (--- to +++)[d]	Struct flexibility (0 to +++)[e]	Construct MW (kDa)	$T_{1/2}$ (h)	Cl (ml/h/kg)	% Dose in urine (day)	% Dose in liver (day)	Ref
		Surface characteristics							PK parameters				
Triazine[h]	2	10 (5)	14	6 (NH_2), 8(OH)	-	0	0	73	100	1	9 (2)	10 (2)	1
Triazine[h]	2	13 (2)	11	3 (NH_2), 8 (OH)	-	0	0	30	43	2.7	10 (2)	12 (2)	1
Triazine[h]	2	14 (0.6)	10	2 (NH_2), 8 (OH)	-	0	0	11	27	4.9	16 (2)	16 (2)	1
Triazine[h]	2	9 (2)	15	3 (NH_2), 12 (drug)	pac	0	0	39[f]	15	14[g]	35 (3)	11 (2)	2
Triazine[h]	2	8 (2)	16	4 (NH_2), 12 (drug)	pac	0	0	37	19	9[g]	55 (3)	22 (2)	2
Triazine[h]	2	6.5 (2)	17.5	5.5 (NH_2), 12 (drug)	pac	0	0	34	20	6[g]	41 (3)	10 (2)	2
Triazine[h]	2	6.5 (5)	17.5	1.5 (NH_2), 16 (drug)	pac	0	0	61	38	1.4	5 (2)	20 (2)	3
PAMAM	5	0	128	128 (OH)	-	0	0	29	3	500[g]	-	12 (1)	4
PAMAM	6	0	256	256 (OH)	-	0	0	58	4	250[g]	-	35 (1)	4
PAMAM	7	0	512	512 (OH)	-	0	0	117	6	50[g]	-	7 (1)	4
PAMAM	5	10 (2)	118	74 (Ac), 44 (NH_2)	-	+	0	52	14[g]	2[g]	2 (2)	15 (2)	5
PAMAM	4	0	64	7 (NH_2), 57 (do3a-Gd)	-	0	0	50	72[g]	13[g]	35 (2)	40(2)	6
PAMAM	5	0	128	10 (DTPA-Tc), 81 (Ac), 9 (biotin), 28 (NH_2)	-	0	0	~41	19[g]*	12[g]	-	57 (0.25)	7
PAMAM	3	12 (5)	12	2 (NH_2), 10 (Do3a-Gd)	-	0	0	69	20	-	-	8	8
PAMAM	3	9 (2)	15	15 (Do3a-Gd)	-	0	0	33	3	-	-	4	8
PAMAM	2	3 (5)	9	9 (Do3a-Gd)	-	0	0	24	0.6	-	38	1	8
PAMAM	2	7 (2)	5	5 (D03a-Gd)	-	0	0	21	6	-	30	7	8
PAMAM	4	60 (5)	4	4 (Ac)	-	0	0	334	78[g]	1[g]	-	6 (1)	9
PAMAM	5	110 (2)	5	5 (Ac)	-	0	0	284	31[g]	3[g]	-	7 (1)	9
PAMAM	4	63 (2)	1	1 (Ac)	-	0	0	162	41[g]	3	-	7(1)	9
PAMAM	4	0	64	64 (Ac)	-	0	0	36	2[g]	36[g]	-	4(1)	9
PAMAM	3	0	32	29 (NH_2), Cy3 (3)	-	+++	0	8	2	40[g]	-	4 (0.25)	10
PAMAM	3	24 (1)	8	5 (NH_2), Cy3 (3)	-	0	0	33	18	3[g]	-	4 (0.25)	10
polyester	3	8 (20)	8	8 (OH)	-	0	+	160	50	2	7(2)	2(0.4)	11
polyester	2	4 (20)	4	4 (OH)	-	0	++	87	25	3	10(2)	2(0.4)	11

polyester	3	8 (10)	8	8 (OH)	-	0	+	85	40	2	2(2)	6(0.4)	11
polyester	3	8 (5)	8	8 (OH)	-	0	+	45	31	3	3(2)	4(0.4)	11
polyester	1	2 (20)	2	2 (OH)	-	0	+++	44	1	152	20(2)	2(0.4)	11
polyester	2	4 (10)	4	4 (OH)	-	0	++	43	26	4	34(2)	6(0.4)	11
polyester	2	4 (5)	4	4 (OH)	-	0	++	23	11	21	22(2)	2(0.4)	11
polyester	1	2 (10)	2	2 (OH)	-	0	+++	22	8	103	33(2)	1(0.4)	11
polyglycerol	2	0	16	16 (OH)	-	0	0	6	16[g]	14[g]	55(0.04)	8(1)	12
polyglycerol	2	0	16	6 (SO_3), 10 (OH)	-	-	0	9	1[g]	56[g]	5(0.04)	90(1)	12
polyglycerol	2	0	16	13 (SO_3), 3 (OH)	-	--	0	13	1[g]	64[g]	1(0.04)	76(1)	12
polylysine	5	32 (1)	32	32 (COOH)	-	-	+	64	33	3	16 (5)	16(5)	13
polylysine	5	28 (1)	36	16 (NH_2), 15 (drug), 5 (CNNH2)	dox	+	+	53	34	2	3(5)	9(5)	14
polylysine	5	28 (1)	36	16 (NH_2), 20 (-CNNH$_2$)	-	+	+	45	22	3	4(5)	4(3)	14
polylysine	5	28 (1)	36	36 (NH_2)	-	++	+	41	29	14	4(3)	37(3)	14
polylysine	5	18 (1)	46	23 (NH_2), 15 (drug), 8 (-CNNH$_2$)	dox	+	+	36	35	2	13(5)	5(5)	14
polylysine	5	18 (1)	46	23 (NH_2), 23 (CNNH$_2$)	-	+	+	31	25	3	16(5)	3(5)	14
polylysine	5	18 (1)	55	55 (NH_2)	-	++	+	27	30	2	24(3)	5(3)	14
polylysine	5	30 (1)	34	4 (NH_2), 15 (-CNNH$_2$), 15 (drug)	dox	0	+	56	51	1	-	-	15
polylysine	5	32 (1)	32	6 (NH_2), 26 (drug)	MTX[otb]	0	0	64	26	2	9 (5)	8(5)	16
polylysine	5	32 (1)	32	6 (NH_2), 26 (drug)	MTX	-	0	64	1	24	2 (3)	52 (3)	16
polylysine	5	32 (1)	32	4 (NH_2), 28 (drug)	MTX[otb]	0	+	71	33	2	14 (5)	10(5)	16
polylysine	5	32 (1)	32	8 (NH_2), 24 (drug)	MTX	-	+	68	0.3	65	2 (3)	85(3)	16
polylysine	4	32 (0.57)	0	0	-	0	0	22	14	9	33 (1)	2 (1)	17
polylysine	4	16 (0.57)	16	16 (drug)	MTX[otb]	0	0	21	0.4	173	29 (1)	1 (1)	17
polylysine	5	64 (0.57)	0	0	-	0	0	48	37	1	6 (5)	8 (5)	17
polylysine	5	32 (0.57)	32	32 (drug)	MTX[otb]	0	0	42	24	5	2 (5)	10(5)	17
polylysine	3	8 (0.57)	8	8 (drug)	MTX[otb]	0	0	11	0.1	443	56 (1)	1 (1)	17
polylysine	3	8 (1)	8	8 (drug)	MTX[otb]	0	0	15	0.2	330	64 (1)	1(1)	17
polylysine	4	16 (1)	16	16 (drug)	MTX[otb]	0	0	30	21	5	24(4)	7(4)	17
polylysine	4	16 (2.3)	16	16 (drug)	MTX[otb]	0	0	47	34	2	8(5)	10(5)	17

polylysine	5	32 (1)	32	32 (drug)	MTX[otb]	0	0	59	51	2	1(7)	12(7)	17
polylysine	4	16 (0.57)	16	16 (NH_2)	-	++	+	13	0.1	213	74 (1)	3(1)	18
polylysine	4	16 (0.57)	16	16 (Ac)	-	0	+	14	0.1	1433	72(1)	0.3(1)	18
polylysine	4	32 (2)	0	0	-	0	+	68	75	1	3(7)	9(7)	19
polylysine	3	16 (2)	0	0	-	0	+	34	24	3	26 (5)	4(5)	19
polylysine	4	32 (0.57)	0	0	-	0	+	22	10	17	43(1)	2(1)	19
polylysine	4	32 (0.2)	0	0	-	0	+	11	0.7	383	80(1)	0(1)	19
polylysine	3	16 (0.2)	0	0	-	0	+	6	0.6	647	82(1)	0(1)	19
polylysine	4	0	32	32 (COOH)	-	--	+	5	0.9	71	25(1)	12(1)	20
polylysine	4	0	16	16 (SO_4)	-	---	+	10	0.9	21	30 (1)	26(1)	20
polylysine	4	0	32	32 (SO_4)	-	---	+	14	1	24	3(1)	49(1)	20
polylysine	4	0	32	32 ($[SO_4]_2$)	-	---	+	7	0.2	1736	63(1)	0(1)	20
polylysine	3	0	16	16 (NH_2)	-	+++	+	2	0.1	1942	8(1)	5(1)	21
polylysine	4	0	32	32 (NH_2)	-	+++	+	4	0.1	4630	4(1)	10(1)	21
polylysine	4	0	32	32 (NH_2)	-	+++	+	4	0.1	2880	4(1)	24(1)	21

[a]Dendrimer generation

[b]Functionality or identity of chemical groups conjugated to non-PEGylated surface reactive sites

[c]Surface conjugated drugs representing paclitaxel (pac), doxorubicin (dox), α-carboxyl OtButylated methotrexate (MTX[otb]) and methotrexate bearing unmodified α-carboxyl functionality (MTX).

[d]Strength of surface charge (from highly anionic [---] to highly cationic [+++]). Assigned based on discussion in the respective manuscripts or based on the number and type of surface charge as well as surface PEG loading.

[e]Structural flexibility of the dendrimer (from relatively rigid [0] to highly flexible [+++]). Relative structural flexibility of each dendrimer construct was assigned based on discussion in the respective manuscripts.

[f]Exists as a 400 kDa aggregate in solution.

[g]Pharmacokinetic parameters calculated based on data that was extrapolated from plasma concentration vs time curves shown in the manuscript.

[h]Surface treatment of the published triazine dendrimers has resulted in 24 available surface groups rather than the standard 16.

*Represents a recalculated value since the value reported in the manuscript was not the correct terminal Half-life.

REFERENCES

1. Lim, J. *et al.* The role of the size and number of polyethylene glycol chains in the biodistribution and tumour localization of triazine dendrimers. *Molecular Pharmaceutics* **5**, 540-547 (2008).
2. Lim, J. *et al.* Design, Synthesis, Characterization, and Biological Evaluation of Triazine Dendrimers Bearing Paclitaxel Using Ester and Ester/Disulfide Linkages. *Bioconjugate Chemistry* **20**, 2154-2161 (2009).
3. Lee, C. *et al.* Design, Synthesis and Biological Assessment of a Triazine Dendrimer with Approximately 16 Paclitaxel Groups and 8 PEG Groups. *Molecular Pharmaceutics* **10**, 4452-4461 (2013).
4. Sadekar, S. *et al.* Comparative Pharmacokinetics of Pamam-Oh Dendrimers and Hpma Copolymers in Ovarian-Tumor-Bearing Mice. *Drug Deliv Transl Res* **3**, 260-271 (2013).
5. Medina, S.H. *et al.* Targeting hepatic cancer cells with pegylated dendrimers displaying N-acetylgalactosamine and SP94 peptide ligands. *Adv Healthc Mater* **2**, 1337-50 (2013).
6. Biricova, V., Laznickova, A., Laznicek, M., Polasek, M. & Hermann, P. Radiolabeling of PAMAM dendrimers conjugated to a pyridine-N-oxide DOTA analog with (1)(1)(1)In: Optimization of reaction conditions and biodistribution. *J Pharm Biomed Anal* **56**, 505-12 (2011).
7. Xu, X. *et al.* Radiosynthesis, biodistribution and micro-SPECT imaging study of dendrimer-avidin conjugate. *Bioorg Med Chem* **19**, 1643-8 (2011).
8. Margerum, L.D. *et al.* Gadolinium(III) DO3A macrocycles and polyethylene glycol coupled to dendrimers - Effect of molecular weight on physical and biological

properties of macromolecular magnetic resonance imaging contrast agents. *Journal of Alloys and Compounds* **249**, 185-190 (1997).

9. Kojima, C., Regino, C., Umeda, Y., Kobayashi, H. & Kono, K. Influence of dendrimer generation and polyethylene glycol length on the biodistribution of PEGylated dendrimers. *Int J Pharm* **383**, 293-396 (2010).

10. Zhong, Q., Merkel, O.M., Reineke, J.J. & da Rocha, S.R. Effect of the Route of Administration and PEGylation of Poly(amidoamine) Dendrimers on Their Systemic and Lung Cellular Biodistribution. *Mol Pharm* (2016).

11. Gillies, E.R., Dy, E., Frechet, J.M. & Szoka, F.C. Biological evaluation of polyester dendrimer: poly(ethylene oxide) "bow-tie" hybrids with tunable molecular weight and architecture. *Mol Pharm* **2**, 129-38 (2005).

12. Pant, K. *et al.* Synthesis and biodistribution studies of (3)H- and (64)Cu-labeled dendritic polyglycerol and dendritic polyglycerol sulfate. *Bioconjug Chem* **26**, 906-18 (2015).

13. Kaminskas, L.M. *et al.* Methotrexate-Conjugated PEGylated Dendrimers Show Differential Patterns of Deposition and Activity in Tumor-Burdened Lymph Nodes after Intravenous and Subcutaneous Administration in Rats. *Molecular Pharmaceutics* **12**, 432-443 (2015).

14. Kaminskas, L.M. *et al.* Characterisation and tumour targeting of PEGylated polylysine dendrimers bearing doxorubicin via a pH labile linker. *J Control Release* **152**, 241-8 (2011).

15. Kaminskas, L.M. *et al.* Doxorubicin-conjugated PEGylated dendrimers show similar tumoricidal activity but lower systemic toxicity when compared to PEGylated liposome and solution formulations in mouse and rat tumor models. *Mol Pharm* **9**, 422-32 (2012).

16. Kaminskas, L.M. *et al.* Capping methotrexate alpha-carboxyl groups enhances systemic exposure and retains the cytotoxicity of drug conjugated PEGylated polylysine dendrimers. *Mol Pharm* **8**, 338-49 (2011).
17. Kaminskas, L.M. *et al.* Pharmacokinetics and tumor disposition of PEGylated, methotrexate conjugated poly-l-lysine dendrimers. *Mol Pharm* **6**, 1190-204 (2009).
18. Kaminskas, L.M. *et al.* Partly-PEGylated Poly-L-lysine Dendrimers Have Reduced Plasma Stability and Circulation Times Compared With Fully PEGylated Dendrimers. *Journal of Pharmaceutical Sciences* **98**, 3871-3875 (2009).
19. Kaminskas, L.M. *et al.* The impact of molecular weight and PEG chain length on the systemic pharmacokinetics of PEGylated poly l-lysine dendrimers. *Mol Pharm* **5**, 449-63 (2008).
20. Kaminskas, L.M. *et al.* Impact of surface derivatization of poly-L-lysine dendrimers with anionic arylsulfonate or succinate groups on intravenous pharmacokinetics and disposition. *Mol Pharm* **4**, 949-61 (2007).
21. Boyd, B.J. *et al.* Cationic poly-L-lysine dendrimers: pharmacokinetics, biodistribution, and evidence for metabolism and bioresorption after intravenous administration to rats. *Mol Pharm* **3**, 614-27 (2006).
